# The Effect of Increased Loads of Dissolved Organic Matter on Estuarine Microbial Community Composition and Function

**DOI:** 10.3389/fmicb.2017.00351

**Published:** 2017-03-09

**Authors:** Sachia J. Traving, Owen Rowe, Nina M. Jakobsen, Helle Sørensen, Julie Dinasquet, Colin A. Stedmon, Agneta Andersson, Lasse Riemann

**Affiliations:** ^1^Centre for Ocean Life, Marine Biological Section, University of CopenhagenHelsingør, Denmark; ^2^Umeå Marine Sciences Centre, Umeå UniversityHörnefors, Sweden; ^3^Department of Ecology and Environmental Science, Umeå UniversityUmeå, Sweden; ^4^Laboratory for Applied Statistics, Department of Mathematical Sciences, University of CopenhagenCopenhagen, Denmark; ^5^Marine Biological Section, University of CopenhagenHelsingør, Denmark; ^6^Centre for Ocean Life, National Institute of Aquatic Resources, Technical University of DenmarkCharlottenlund, Denmark

**Keywords:** bacterioplankton community composition, community functions, extracellular enzymes, 16S rRNA, climate change, dissolved organic matter, generalized linear models, Baltic Sea

## Abstract

Increased river loads are projected as one of the major consequences of climate change in the northern hemisphere, leading to elevated inputs of riverine dissolved organic matter (DOM) and inorganic nutrients to coastal ecosystems. The objective of this study was to investigate the effects of elevated DOM on a coastal pelagic food web from the coastal northern Baltic Sea, in a 32-day mesocosm experiment. In particular, the study addresses the response of bacterioplankton to differences in character and composition of supplied DOM. The supplied DOM differed in stoichiometry and quality and had pronounced effects on the recipient bacterioplankton, driving compositional changes in response to DOM type. The shifts in bacterioplankton community composition were especially driven by the proliferation of Bacteroidetes, Gemmatimonadetes, Planctomycetes, and Alpha- and Betaproteobacteria populations. The DOM additions stimulated protease activity and a release of inorganic nutrients, suggesting that DOM was actively processed. However, no difference between DOM types was detected in these functions despite different community compositions. Extensive release of re-mineralized carbon, nitrogen and phosphorus was associated with the bacterial processing, corresponding to 25–85% of the supplied DOM. The DOM additions had a negative effect on phytoplankton with decreased Chl *a* and biomass, particularly during the first half of the experiment. However, the accumulating nutrients likely stimulated phytoplankton biomass which was observed to increase towards the end of the experiment. This suggests that the nutrient access partially outweighed the negative effect of increased light attenuation by accumulating DOM. Taken together, our experimental data suggest that parts of the future elevated riverine DOM supply to the Baltic Sea will be efficiently mineralized by microbes. This will have consequences for bacterioplankton and phytoplankton community composition and function, and significantly affect nutrient biogeochemistry.

## Introduction

Climate change is projected to increase precipitation in the northern hemisphere, by as much as 30% in the Baltic region (Andersson et al., 2015). This will lead to a 15–20% increase in freshwater runoff (Meier et al., 2012) and parallel increases in loadings of riverine dissolved organic matter (DOM) and nutrients to coastal systems. The input of DOM and nutrients will conceivably affect the composition, function and activity of the recipient microbial communities and thereby impact the entire ecosystem (Sandberg et al., 2004; Wikner and Andersson, 2012). The elevated riverine DOM supply will likely increase bacterial respiration, as humic material is typically carbon rich and fuels bacterial respiration (Benner, 2003; Fasching et al., 2014). In coastal systems riverine DOM may support a significant fraction of bacterioplankton activity, thereby decoupling the activity of heterotrophic bacteria from phytoplankton production (Kemp et al., 1997; Sandberg et al., 2004; Wikner and Andersson, 2012; Figueroa et al., 2016). Moreover, an increased allochthonous carbon load supporting bacterial activity may lead to increased competition for inorganic nutrients between bacteria and phytoplankton (Thingstad et al., 2008; Wikner and Andersson, 2012), which may further weaken the link between phytoplankton production and bacterial activity and expand zones of net heterotrophy.

Bacterial communities are tightly coupled to the concentration and composition of DOM, with shifts in DOM composition inducing changes in bacterial community composition and functionality (e.g., Gasol et al., 2002; Kritzberg et al., 2004; Judd et al., 2006; Alonso-Sáez and Gasol, 2007). Therefore, the concentration and characteristics of riverine DOM may be important for the ultimate fate of this additional organic matter, such as the partitioning between local remineralization, passage through the food web or export to adjacent shelf seas. In addition, the increases in DOM will likely lead to “brownification” of the recipient waters and increased light attenuation due to higher concentrations of humic substances in DOM (Roulet and Moore, 2006), which is commonly observed in coastal regions (Sanden and Håkansson, 1996; Aksnes and Ohman, 2009; Frigstad et al., 2013). This may affect phytoplankton biomass and productivity ultimately leading to carbon limitation of bacterioplankton growth (Thingstad et al., 2008).

The Baltic Sea consists of basins characterized by distinct hydrology and differing land usage. The consequences of climate change are therefore expected to vary regionally (Rönnberg and Bonsdorff, 2004). The north receives large inputs of riverine DOM rich in humic substances (Andersson et al., 2015), while the south receives a comparatively nutrient rich riverine DOM inflow (Stepanauskas et al., 2002). The riverine loadings differ significantly in C:N:P stoichiometry depending on region and can potentially have very different effects on the recipient ecosystem. However, it remains unclear how the character and concentration of DOM supplied by rivers will influence the bacterioplankton and thereby the fate of carbon and nutrients in the system.

The objective of this study was to experimentally investigate the effects of elevated DOM on a recipient coastal microbial community and examine the impact on functional activity and population structures. Specifically, we focused on the response of the microbial community to differences in DOM characteristics.

## Materials and Methods

### Collection Site and Mesocosm Setup

The experiment was performed from May 21–June 25 2012 at Umeå Marine Sciences Centre (UMF), Sweden, in 12 indoor mesocosm tanks of 4.87 m height (water column) and a diameter of 0.76 m. The water was collected on May 21 and 22 from a regularly sampled station in the Bothnian Sea (63°32′05.2″ N 19°56′09.6″ E) at 4 m depth, using a rotating pump (Flygt DS 3057.181 MT-230), with a 1.5 mm mesh pre-filter. The salinity of the water was 3.8 (Seaguard CTD, Aanderaa) and *in situ* temperature was 6°C. The water was transported in 1 m^3^ polythene containers to the field station and thereafter carefully pumped into the mesocosms, ensuring an equal distribution of the water between tanks. All mesocosms received inorganic nutrients on May 21, at concentrations of 0.7 μmol l^-1^ nitrogen and 0.09 μmol l^-1^ phosphorus, to prevent nutrient exhausting during a 4 days acclimation period. During this period the temperature was incrementally increased to 15°C. To prevent stratification, a constant and gentle bubbling was applied at 0.6 m depth and convective stirring was generated by maintaining the upper section of each mesocosm tank at 14.8°C, the middle section at 15.0°C, and the lower section at 15.2°C, the latter supported by a 250 W electrical heater. Light was provided on a 12 h light:dark cycle, using 150 W halogen lamps (MASTERColour CDM-T 150W/942 G12 1CT. Phillips, yielding a photosynthetically active radiation (PAR) light level of ∼400 μmol photons m^-2^ s^-1^ immediately below the surface.

### Treatments

The 12 mesocosm tanks received one of four treatments, each replicated in triplicate. Two treatments (North and South) received additions of soil extracted DOM (**Table 1**), prepared from soil samples collected along the riverbanks of two contrasting rivers that discharge into the Baltic Sea. The North treatment was collected from the Öre river in northern Sweden (63°32′40.7″ N 19°42′35.6″ E), the catchment area of this river being predominantly characterized by coniferous and deciduous forest. The South treatment was collected from the riverbanks of the Reda river, Poland (54°38′35.80″ N, 18°27′41.28″ E), with a catchment area characterized by agricultural activities and some broad-leaf forest. Soil extracts were prepared and stored according to the procedure described in Lefebure et al. (2013). In brief, soil extracts were mixed with Milli-Q water and ion exchange resin (Amberlite IRC 7481) for 48 h at 4°C, and then filtered through a 90 μm mesh. The carbon (C), nitrogen (N), and phosphorus (P) content of the extracts were determined using a Shimadzu TOC-5000 carbon analyzer and a Braan and Luebbe TRAACS 800 autoanalyzer. The experiment was designed to simulate future predictions of increased riverine DOM load to the Baltic (Eriksson Hägg et al., 2010). The DOM treatments aimed to increase dissolved organic carbon (DOC) in the water with roughly 50% (North) and 100% (South) relative to the DOC concentration (∼333 μmol l^-1^) in the Bothnian Sea. Two additional treatments were set up as controls for the DOM addition, receiving only inorganic N and P (**Table 1**), corresponding to the inorganic N and P concentrations in the North and South, from here on referred to as “Ctrl_N_” and “Ctrl_S_,” respectively.

**Table 1:** Daily supplements in carbon (C), nitrogen (N), and phosphorus (P) to the mesocosms after addition of soil extracted dissolved organic matter.

Treatment	C μmol l^-1^	N μmol l^-1^	P μmol l^-1^
North	10 (83)	1.1 (8.8)	0.03 (0.2)
South	20 (166)	2.3 (20)	0.13 (1.1)
Ctrl_N_	–	0.001 (0.01)	0.0008 (0.007)
Ctrl_S_	–	0.009 (0.08)	0.005 (0.04)

*In parentheses, the increase after the initial ∼22% addition; Section “Materials and Methods.” Ctrl_*N*_ and Ctrl_*S*_ received inorganic N and P in the stoichiometry and concentrations supplied with the DOM in the North and South treatments.*

Three days prior to first sampling DOM treatments received an initial large DOM addition, corresponding to ∼22% of the total amount of DOC added during the experiment, and this was then followed by smaller daily doses (**Table 1**). Control treatments also received corresponding N:P additions. The experiment had a duration of 32 days and sampling was initiated on May the 28th by the addition of seven equally sized young-of-the-year perch (*Perca fluviatilis*) to each mesocosm. The fish were added in order to complete a pelagic food web, encompassing bacteria to zooplanktivorous fish. Every second day 40 l of water from 2 m depth was removed, and replaced with 40 l of 0.2 μm filtered Bothnian Sea water pumped into the marine research station from a point ∼2 km offshore (63°33′15.6″ N 19°50′08.4″ E). Samples were collected on days: 0, 3, 7, 10, 14, 17, 21, 24, and 28. The majority of parameters were sampled/processed on all days and extracellular enzymes were measured on two additional occasions, days 5 and 12.

### Water Chemistry, Phytoplankton Biomass, and Community Composition

Colored dissolved organic matter (CDOM) samples were filtered through pre-combusted Whatman^®^ GF/F filters, and the filtrate was immediately frozen at -20°C until further processing. Samples were measured within 2 months of the experiment on a Horiba Scientific Aqualog according to Murphy et al. (2011). CDOM absorption properties were characterized by calculating the spectral slope across 300–650 nm (Stedmon et al., 2000). Samples for inorganic nutrients and DOC were filtered through 0.22 μm cellulose-acetate filters (Gelman Supor^®^) and immediately measured. Inorganic nutrients (NH_4_^+^, NO_3_^-^, NO_2_^-^, and PO_4_^3-^) were measured in whole water samples using continuous flow analysis on a Quaatro system (Seal Analytical), following methods outlined in Grasshoff et al. (1983) and Helcom guidelines (HELCOM, 2014). Samples for measurement of total dissolved nitrogen (TDN), total dissolved phosphorus (TDP), and DOC were filtered through 0.2 μm Acrodisk (Supor^®^) filters (Pall Corporation). TDN and TDP samples were subjected to oxidative digestion using the peroxodisulfate/boric acid system (Koroleff, 1983), and then analyzed using the same method as for nitrate and phosphate. DOC was approximated as Non Purgeable Organic Carbon and analyzed using high temperature catalytic oxidation followed by non-dispersive infrared sensor detection of the gaseous CO_2_. The instrument used was a Shimadzu TOC-L Total Organic Carbon Analyzer (Shimadzu Corporation). Dissolved organic nitrogen (DON) was calculated by subtracting NH_4_^+^, NO_3_^-^, NO_2_^-^ from TDN, and total dissolved phosphorus (DOP) was calculated by subtracting PO_4_^3-^ from TDP.

Chlorophyll *a* (Chl *a*) and phytoplankton community composition were measured as detailed in Lefebure et al. (2013). In brief, Chl *a* samples were filtered onto Whatman^®^ GF/F filters (100 ml in duplicates), extracted in 95% ethanol for 24 h in the dark, and measured on a Perkin Elmer LS 30 fluorometer. Samples for phytoplankton community composition were preserved in Lugol’s solution and later counted using microscopy and converted to biomass (μg C l^-1^) using carbon conversion (Menden-Deuer and Lessard, 2000).

### Bacterial Functions

Bacterial production was assayed using ^3^H-thymidine incorporation (Fuhrman and Azam, 1982). Samples were incubated with thymidine (final concentration of 24 nM, and an activity of 84.4 Ci mmol^-1^) at 15°C for ∼1 h, and analyzed using a Beckman 6500 scintillation counter. Bacterial production was estimated using a conversion factor of 1.4 × 10^18^ cells μmol^-1^ thymidine (Wikner and Hagström, 1999) and 20.4 fg C cell^-1^ (Lee and Fuhrman, 1987). Samples for bacterial abundance were preserved in a 4% final concentration of formaldehyde, stained with acridine orange, and enumerated using an epifluorescence microscope (Zeiss Axiovert 100, Thornwood, NY, USA) and image analysis (Blackburn et al., 1998).

Extracellular enzyme activities were assayed using fluorogenic 4-methylumbelliferone (MUF) and 7-amino-4-methylcoumarin (MCA) substrates (Sigma-Aldrich, St. Louis, MO, USA). The enzyme assays were prepared according to Hoppe (1983), modified to a reaction volume of 200 μl with 400 μM substrate (final concentration). The enzymes assayed and substrates used were: protease (L-Leucine-MCA), alkaline phosphatase (MUF-phosphate disodium salt), β-NAGase (MUF-*N*-acetyl-β-D-glucosaminide), lipase (MUF-oleate) and α- and β-glucosidases (MUF-α-D-glycopyranoside and MUF-β-D-glycopyranoside. respectively). Assays were measured in triplicates at 355 nm excitation and 460 nm emission on a FLUOstar OPTIMA plate reader (BMG, Labtech GmbH, Ortenberg, Germany). Assays were incubated at 15°C in the dark and followed for 5 h.

### Bacterial Community – Extraction of Nucleic Acids and 16S rRNA Amplicon Sequencing

Samples (500 ml) for DNA and RNA extraction were filtered onto separate 0.22 μm cellulose-acetate filters (Gelman Supor^®^), except for the South treatment which clogged at 300 ml. DNA filters were stored in 1 ml Tris-EDTA buffer (VWR, Radnor, PA, USA) at -20°C, while RNA filters were covered with 1 ml RNA*later*^®^ (Ambion^®^, Life Technologies, Carlsbad, CA, USA), flash frozen in liquid nitrogen, and stored at -80°C until further processing. Extractions of DNA and RNA were done using the E.Z.N.A^®^ Tissue DNA kit (OMEGA bio-tek, USA) and PowerWater^®^ RNA isolation kit (MO BIO Laboratories, Inc., USA), respectively. Synthesis of cDNA was done according to Bentzon-Tilia et al. (2015) using TaqMan reverse-transcription reagents (Applied Biosystems, Foster City, CA, USA) and the 806r primer listed below. 16S ribosomal RNA (cDNA) and DNA amplicons of the V4 region of bacterial and archaeal communities were obtained using the primers 515f (5′-126 GTGCCAGCMGCCGCGGTAA) and 806r (5′-GGACTACHVGGGTWTCTAAT) (Caporaso et al., 2012). Polymerase chain reactions (PCRs) were performed in 25 μl reaction volume containing 1 (DNA) and 10 (cDNA) ng template, primers, MyTaq DNA polymerase reagents (Saveen & Verner AB, Limhamn, Sweden), 0.07 nmol BSA (Sigma-Aldrich, St. Louis, MO, USA) and 2 nmol MgCl_2_ (DNA Diagnostic, Risskov, Denmark). The PCR conditions included an initial denaturing step at 94°C for 3 min followed by 29 cycles of 94°C for 45 s, 50°C for 1 min, 72°C for 1 min 30 s, and a final step of elongation at 72°C for 10 min. Triplicate PCR reactions were pooled for each sample, purified using the Agencourt AMPure XP purification kit (Beckman Coulter, Inc., Brea, CA, USA), and quantified using the Quant-iT^TM^ PicoGreen^®^ quantification kit (Invitrogen, Waltham, MA, USA) and a FLUOstar OPTIMA plate reader (BMG Labtech GmbH, Ortenberg, Germany). PCR amplicons were pooled at equimolar concentrations and submitted for commercial paired-end sequencing on an Illumina MiSeq.

Sequence reads were assembled, trimmed to a mean length of 252 nucleotides, and de-multiplexed using QIIME v1.9 (Caporaso et al., 2010). Removal of singletons and clustering of operational taxonomic units (OTUs) at 97% similarity was done in USEARCH v1.8 (Edgar, 2010) using the UPARSE-OTU algorithm (Edgar, 2013) with implicit chimera check. Taxonomy was assigned in QIIME using uclust (Edgar, 2010) and the Greengenes v.13.8 reference database (McDonald et al., 2012). Chloroplasts and mitochondrial reads were removed before downstream analysis. OTUs only occurring once in the dataset and/or including < 10 reads in total were excluded. Henceforth, 16S rRNA and rRNA gene amplicons are referred to as rRNA and rDNA, respectively. Sequences were deposited in GenBank NCBI (accession numbers KX178204-KX179464). In the rRNA dataset three samples were lost during processing – (day, tank): (21, 4), (17, 5), (28, 10).

### Data Analysis

All data and statistical analyses were carried out in R (R Core Team, 2016) using the R packages mvabund v. 3.11.7 (Wang et al., 2012), pgirmess v. 1.6.4 (Giraudoux, 2016), FactoMineR v. 1.31.4 (Husson et al., 2015), and vegan v. 2.3-2 (Oksanen et al., 2015). For testing differences between treatments, linear mixed models were used; with day, treatment, and their interaction as fixed effects and mesocosm tank as a random effect. Data were log-transformed if appropriate. The average outcome over time was compared for North and Ctrl_N_, South and Ctrl_S_, and for North and South. A total of 45 tests were carried out, and the *p*-values were Bonferroni–Holm adjusted in order to ensure a family wise error rate of at most 5%. Patterns of bacterial community compositions were analyzed using Bray–Curtis distances and visualized by non-metric multidimensional scaling (NMDS). Specifically for the NMDS and descriptions of relative composition of communities, samples were subsampled to a depth of 2280 reads per sample to accommodate for the smallest sample in the set.

Generalized linear models (GLM) were applied to identify the OTUs contributing most to the differences between the four treatments at the beginning (day 0) and end (day 28) of the experiment. The aim of the GLM approach described here is similar to that of the SIMilarity PERcentages analysis (SIMPER, Clarke, 1993), but also takes the mean-variance relationship in the rDNA and rRNA data, into account (Warton et al., 2012). For this analysis, datasets were filtered to include only OTUs with a relative abundance > 1% in at least one observation, leaving 87 and 95 OTUs in the rDNA and rRNA datasets, respectively. For each of these OTUs, and separately for days 0 and 28, univariate negative binomial regression models were fitted with abundance as the response variable. In each model, treatment was included as an explanatory variable while the logarithm of the total abundance of OTUs in each observation was included as an offset variable, in order to take into account the varying sample sizes. The models were fitted using a log link function and assuming an unknown over-dispersion parameter in each model, which was estimated from the data. Likelihood ratio (LR) test statistics for the hypothesis of no treatment effect were computed for each of the univariate models. Corresponding *p*-values adjusted for the multiple tests performed within the rDNA and rRNA datasets, respectively, for each of the days 0 and 28, were computed by means of a step-down resampling procedure using residual permutation (as implemented in Wang et al., 2012). These *p*-values were further adjusted by multiplication with a factor four (a Bonferroni-type correction) to ensure a family-wise error rate of <5% among all tests pertaining to the GLM models. OTUs with the largest LR test statistics are interpreted as the OTUs that exhibit the most notable difference in relative abundance between treatments. For an adjusted *p*-value of less than 0.05, the difference between treatments is considered statistically significant.

## Results

### DOM and Nutrients

**FIGURE 1:**
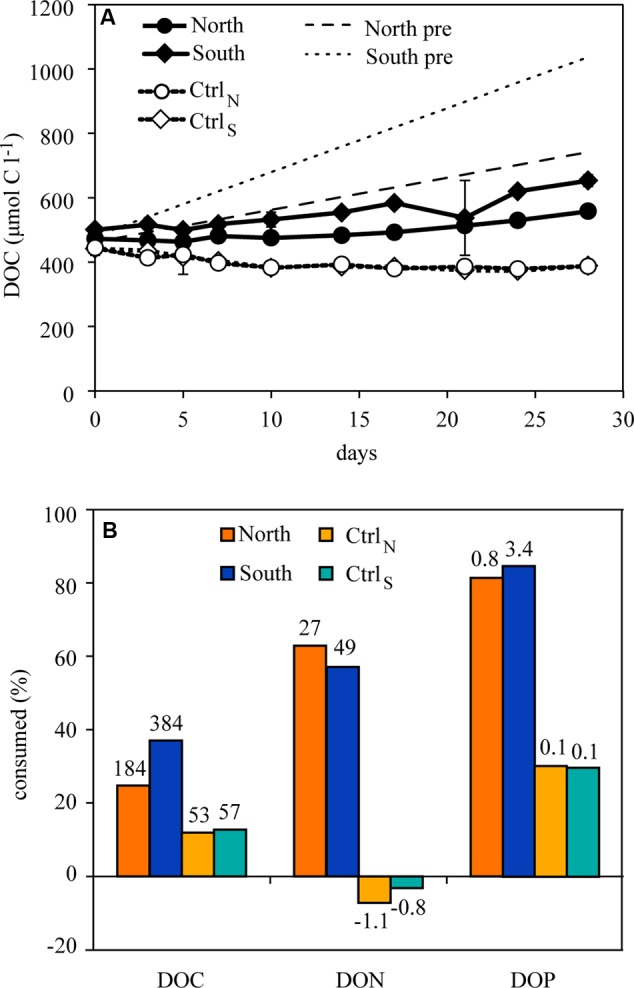
**(A)** Concentrations of dissolved organic carbon (DOC) over time in the four treatments. The dotted lines (“North pre” and “South pre”) represent predictions of accumulated DOC calculated from the respective DOM additions assuming no biological consumption, in North and South, respectively. Error bars represent standard deviations (*n* = 3). **(B)** The stoichiometry of the consumed DOM in the treatments. In the DOM treatments, consumption of DOC is calculated as % difference between the predicted DOC accumulation and measured DOC concentrations, and in the controls the consumption is calculated as the difference between measured start and end concentrations. Consumption of dissolved organic nitrogen (DON) and phosphorus (DOP) was calculated as for DOC. Values above each bar are the consumed concentrations (μmol l^-1^).

Dissolved organic carbon increased significantly during the experiment (**Figure 1A**) in both North and South compared to their controls (adjusted *p* << 0.0001) and also differed between North and South (adjusted *p* < 0.002). However, the increase was less than expected based on the added DOC (**Figure 1A**). The total amount of DOC removed corresponded to 25 and 37% of the added DOC in the North and South treatments, respectively (**Figure 1B**), calculated as the difference between added and measured DOC. In comparison, 12 and 13% of the DOC was removed in the Ctrl_N_ and Ctrl_S_, respectively (calculated as the difference between measured DOC at start and end). For DON and DOP, the communities in both treatments appeared to effectively mineralize a large fraction. In the North treatment 63 and 81% of the added DON and DOP, respectively, was removed and similar high removal was observed in the South treatment (57 and 85% DON and DOP, respectively), despite the higher DOM additions in South. Small amounts of DON accumulated in Ctrl_N_ and Ctrl_S_, corresponding to 7 and 3%, respectively, while 30% of the DOP was removed. The C:N:P stoichiometry of the removed DOM was 239:36:1 and 114:15:1 for North and South, respectively. This corresponded roughly to the stoichiometry of the added DOM, 387:41:1 for North and 156:18:1 in South.

**FIGURE 2:**
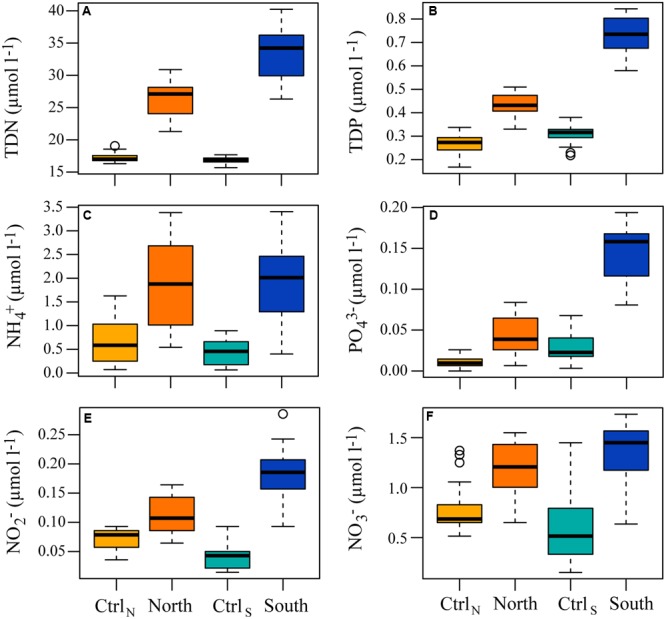
**Box and whisker plots indicating the median concentrations for the whole experiment, with the lower 25th and upper 75th percentile, of total dissolved (A)** nitrogen (TDN), **(B)** phosphorus (TDP), **(C)** NH_4_^+^, **(D)** PO_4_^3-^, **(E)** NO_2_^-^, and **(F)** NO_3_^-^ in the four treatments.

Inorganic nutrients were also affected by the DOM additions, which caused significantly higher concentration of NH_4_^+^ (adjusted *p* < 0.02), NO_3_^-^ (adjusted *p* < 0.001), total N (adjusted *p* << 0.0001), and total P (adjusted *p* << 0.0001) in North and South, when compared to their respective controls (**Figure 2**). In addition, NO_2_^-^ (adjusted *p* < 0.001) and PO_4_^3-^ (adjusted *p* < 0.0001) were also significantly higher in South compared to Ctrl_S_, an effect not detected in the North treatment. Between North and South PO_4_^3-^, total N and P (adjusted *p* < 0.0001) were significantly different.

**FIGURE 3:**
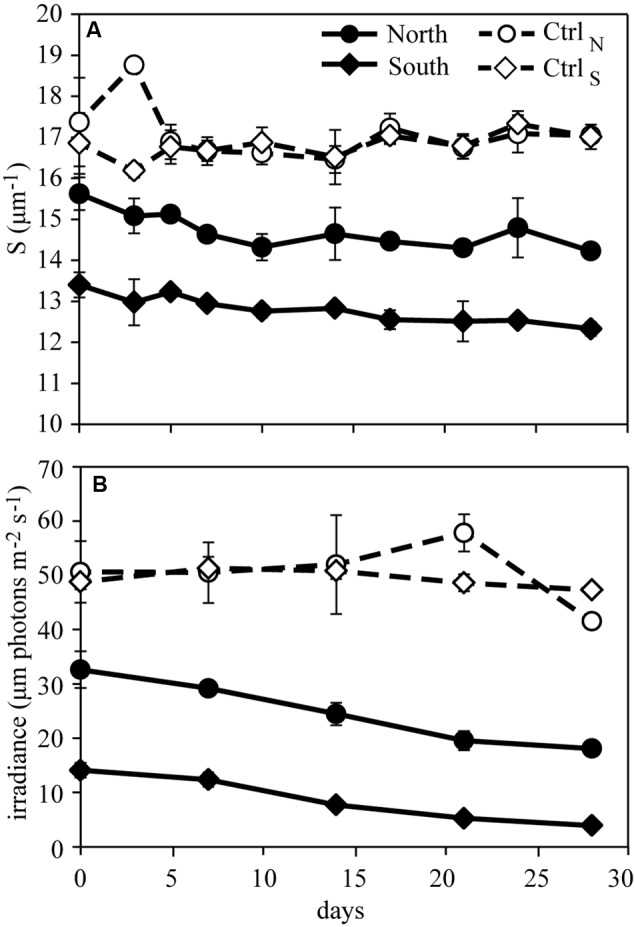
**Development in optical properties during the experiment. (A)** the spectral slope (300–650 nm) of CDOM indicating differences in DOM character between treatments and **(B)** the irradiance of PAR indicating the influence of DOM additions on light penetration. The two controls Ctrl_N_ and Ctrl_S_ remained relatively stable over time. Error bars represent the standard deviations (*n* = 3).

The CDOM slope (S) for the UVA-visible wavelength range (300–650 nm) was used as an indicator of differences in DOM quality between treatments. The UVA-visible range was used due to the direct link between organic matter and light attenuation. Other CDOM parameters measured behaved as expected based on the S values, e.g., short wavelength slopes correlated positively with S, and SUVA was inversely correlated to S. Hence, these were not included in further analyses. For both DOM treatments the slopes decreased with time while the controls showed little systematic change and no noteworthy differences. The DOM additions resulted in lower S values with the lowest values in South (**Figure 3A**). The increases in CDOM absorption over time in the DOM treatments resulted in decreasing PAR levels at 1 m depth during the experiment (**Figure 3B**).

### Phyto-and protozooplankton Biomass and Community Composition

**FIGURE 4:**
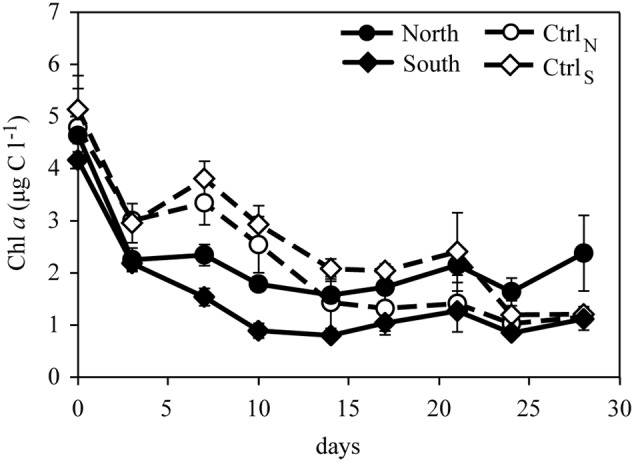
**Concentration of Chl *a* over time in the four treatments.** Error bars represent standard deviation (*n* = 3).

**FIGURE 5:**
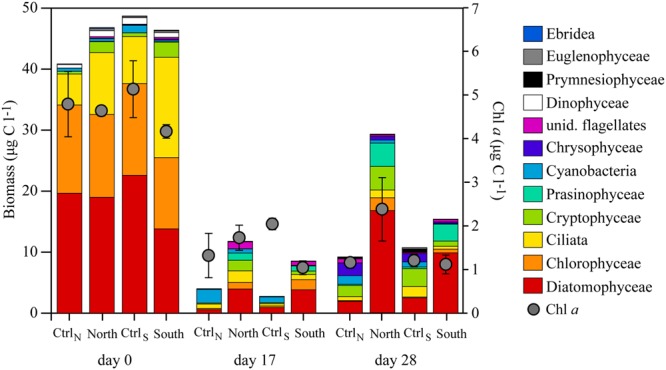
**Phyto- and protozooplankton community composition shown as stacked bars, indicating the total biomass.** Chl *a* is indicated by gray dots. All values are averages from the triplicate mesocosms.

Concentrations of Chl *a* and phytoplankton biomass decreased over time in all treatments accompanied by shifts in phytoplankton community composition (**Figures 4, 5**). Initially diatoms, chlorophytes and ciliates made up the majority of the community in all treatments. On day 17, Ctrl_N_ and Ctrl_S_ were dominated by cyanobacteria whereas North and South were dominated by cryptophytes, prasinophytes and a group of unidentified flagellates. On day 28, total biomass had increased again being highest in North and South treatments with diatoms, chlorophytes and prasinophytes being the dominant groups, whereas chrysophytes, prymnesiophytes, and cyanobacteria dominated in the control treatments.

### Bacterial Response to DOM Additions

**FIGURE 6:**
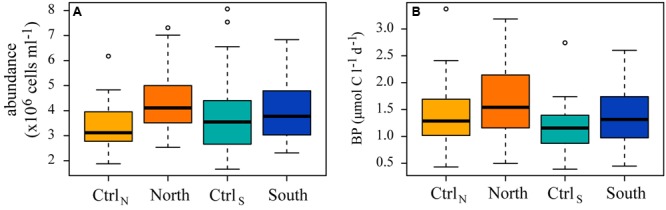
**Box and whisker plots indicating the median (A)** bacterial abundance and **(B)** production (BP) for the whole experiment, with the lower 25th and upper 75th percentile.

Patterns of bacterial abundance and production followed similar trends in the four treatments (**Figure 6**), with no significant differences in the bacterial abundance or production between DOM treatments, and compared to their respective controls.

**FIGURE 7:**
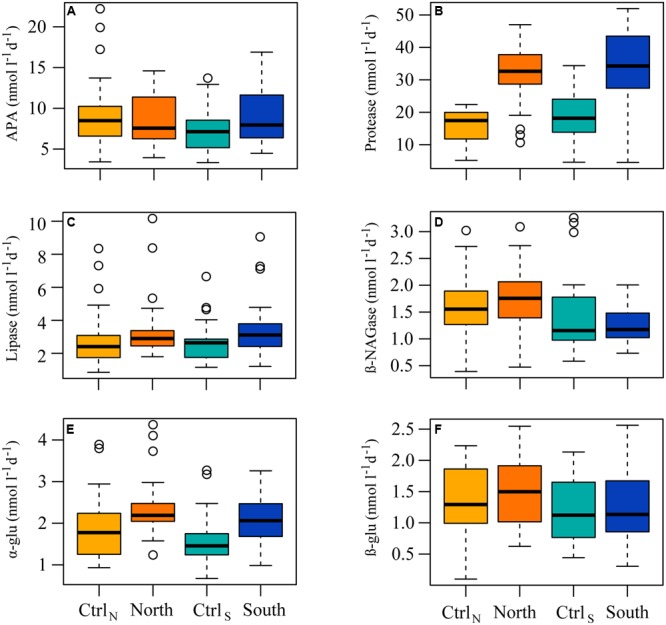
**Box and whisker plots indicating the median rates for the whole experiment, with the lower 25th and upper 75th percentile, of the enzymatic activity of (A)** alkaline phosphatase (APA), **(B)** protease, **(C)** lipase, **(D)** β-NAGase, **(E)** α-glucosidase (α-glu), and **(F)** β-glucosidase (β-glu) in the four treatments.

Extracellular enzyme activities responded to DOM additions, but only protease had significantly higher activity (adjusted *p* < 0.0002), in North and South, compared to their controls (**Figure 7**). Furthermore, protease activity was not significantly different between North and South.

### Shifts in Bacterial Community Composition

**FIGURE 8:**
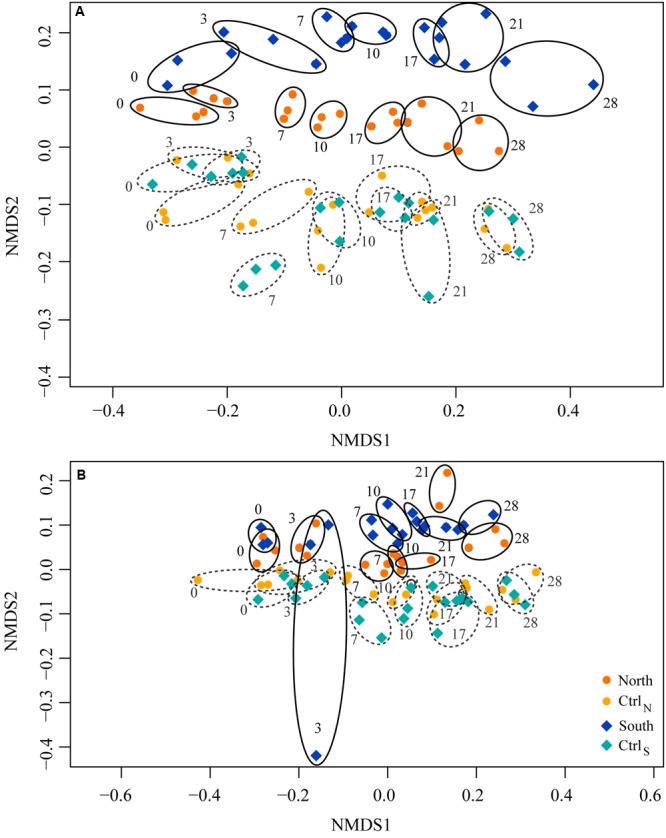
**Non-metric multidimensional scaling (NMDS) plots of changes in (A)** total (rDNA) and **(B)** active (rRNA) community composition in the four treatments over time. Similarities were based on the Bray–Curtis distance. Rings indicate replicate communities from the same day. Numbers indicate days of sampling.

A total of 1,866,318 high quality reads remained after quality and chimera check, with 1,637 reads assigned to *Archaea*. Clustering at 97% similarity resulted in a total of 1,261 OTUs. The composition in the total community (rDNA) changed in response to treatment and time, becoming more dissimilar over time and resulting in North and South communities shifting apart from each other, and from the two controls (**Figure 8A**). A similar shift in composition was observed in the active community (**Figure 8B**).

**FIGURE 9:**
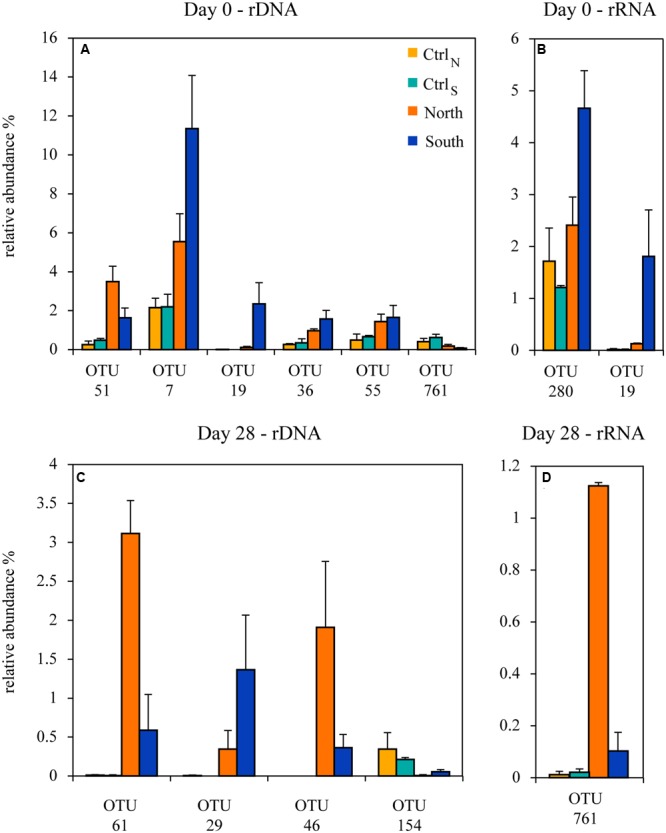
**The relative abundance of operational taxonomic units (OTUs) from the GLM models which contributed significantly to the difference between the four treatments, on day 0 in the (A)** total and **(B)** active communities, and at day 28 in the **(C)** total and **(D)** active communities. Error bars represent standard deviation (*n* = 3).

To further investigate the observed relative changes in community composition, a GLM analysis was applied to identify the OTUs for which relative abundance differed between the four treatments. This was done for the beginning (day 0) and the end (day 28) of the experiment. The OTUs with the most notable and significant differences between treatments, as indicated by the size of their LR test statistics and adjusted *p*-values, are reported together with their relative abundances (**Table 2** and **Figure 9**). At day 0 in the total communities the relative abundance of six OTUs were significantly different between treatments, five Bacteroidetes OTUs (OTU_19, 36, 51, 55, and 761) and one Alphaproteobacteria (OTU_7). In the active communities at day 0, two OTUs (OTU_280 and 19, from Betaproteobacteria and Bacteroidetes, respectively) exhibited significant differences between treatments.

**Table 2:** A list of the operational taxonomic units (OTUs) that contributed significantly to the community composition differences between the four treatments, ordered according to the size of their likelihood-ratio (LR) test statistics and adjusted *p*-value from the generalized linear model (GLM) analyses.

OTU#	Phylum	Taxon	LR statistics	Adjusted *p*-value
**Day 0 rDNA**				
OTU_51	Bacteroidetes	*Flavobacterium*	44.521	0.001
OTU_7	Alphaproteobacteria	*Rhodobacter*	40.985	0.002
OTU_19	Bacteroidetes	Flavobacteriaceae	39.757	0.002
OTU_36	Bacteroidetes	Flavobacteriaceae	33.300	0.004
OTU_55	Bacteroidetes	Flavobacteriales	29.159	0.021
OTU_761	Bacteroidetes	*Fluviicola*	27.911	0.028
**Day 0 rRNA**				
OTU_280	Betaproteobacteria	*Delftia*	34.690	0.003
OTU_19	Bacteroidetes	Flavobacteriaceae	33.440	0.005
**Day 28 rDNA**				
OTU_61	Gemmatimonadetes	KD8-87	38.773	0.003
OTU_29	Bacteroidetes	Sphingobacteriales	36.143	0.004
OTU_46	Gemmatimonadetes	*Gemmatimonas*	35.273	0.006
OTU_154	Planctomycetes	*Pirellulaceae*	30.669	0.038
**Day 28 rRNA**				
OTU_761	Bacteroidetes	*Fluviicola*	31.744	0.025

*Only OTUs which were significant are reported here. Taxonomy was assigned using Greengenes v. 13.8.*

By the end of the experiment the community composition had changed, and the OTUs showing significant differences between treatments in the GLM models had also changed. On day 28, four OTUs were significant in the total communities, two OTUs from Gemmatimonadetes (OTU_46 and 61) and two OTUs from Bacteroidetes and Planctomycetes (OTU_ 29 and 154, respectively). In the active communities on day 28 only a single OTU from Bacteroidetes (OTU_761) showed a significant difference between treatments.

## Discussion

Elevated inputs of riverine DOM have been predicted for the future Baltic Sea (Andersson et al., 2015). The present experiment suggests that a fraction of the terrestrial DOM supplied was available to the microbial community over timescales similar to that of mixing in coastal waters. The organic matter addition was processed by the microbial community and resulted in an accumulation of inorganic nutrients, and this bacterioplankton mediated change in resource utilization stimulated phytoplankton biomass despite the simultaneous decrease in light penetration due to light absorption by CDOM.

### Fate of DOM

Dissolved organic matter flocculation can be an important removal process in estuarine systems (Sholkovitz, 1976; Sholkovitz et al., 1978). However, the majority of studies suggest that this is highly salinity-dependent. DOM flocculation is low at salinities < 5 and a minor factor in systems with relatively stable salinities (Sholkovitz et al., 1978; Søndergaard et al., 2003). As salinities were constant at 3.8 in this experiment it is unlikely that abiotic flocculation contributed significantly to DOM removal. Microbial activity has also been suggested to contribute to DOC flocculation in freshwater with estimated removal rates between 0.3 and 1.2 μmol C l^-1^ d^-1^ (Wachenfeldt et al., 2009). That would correspond to flocculation causing a C removal of 2 to 18% in our experiment, a relatively small fraction of the total C removed from the North and South treatments. We therefore interpret the removed DOM in the mesocosms to be primarily caused by microbial activity. The microbial communities in the DOM treatments consumed organic resources in ratios reflecting the C, N, P – stoichiometry of the input DOM, suggesting a relatively flexible consumption, as observed in bacterial isolates and natural aquatic assemblages (Makino et al., 2003; Godwin and Cotner, 2015). The observed differences in bacterial processing of DOM did not translate into differences in bacterial production or abundances between treatments. This suggests that the additional DOM consumption in North and South was not channeled into the bacterial biomass. However, grazing by hetero- and mixotrophic protists and viral lysis are the major processes controlling bacterial removal, unfortunately neither group was quantified so the fate of the DOM that was channeled into the bacterial commmunity remains speculative.

As a consequence of bacterial degradation the stoichiometry of the input DOM will affect the resource landscape of the recipient water due to the flexible consumption of the bacterioplankton. This seems to be supported by the significantly higher inorganic nutrient concentrations in the DOM treatments compared to the controls, likely causing the increased phytoplankton biomass towards the end of the experiment. The accumulated DOM, i.e., the fraction not utilized by bacteria, was rich in organic C, causing an increased coloration of the water and decreasing levels of PAR, and the CDOM characteristics (S values) in the DOM treatments were lower, typical of terrestrial DOM (Stedmon et al., 2000). In our experiment, the increased light attenuation likely opposed more extensive phytoplankton growth induced by the available re-mineralized inorganic nutrients. Hence, a future elevated outflow of DOM may affect the relative importance of inorganic nutrients and light in the Baltic Sea – the two main factors controlling local phytoplankton growth and structure (Andersson et al., 1996).

The DOM additions stimulated protease activity with significantly higher activities measured in the presence of the terrestrial DOM. This is similar to findings in previous studies demonstrating strong protease stimulation by humic-rich DOM (Stepanauskas et al., 1999). The increased protease activity was consistent with the efficient consumption of DON, and lead to a release and accumulation of inorganic nitrogen. Alkaline phosphatase activity was not significantly different between treatments suggesting that the microbial communities in all four treatments were deficient in P. Indeed total P concentrations in all four treatments were relatively low (<0.9 μmol l^-1^) with more than 80% bound in the organic fraction. Extracellular phosphatases are expressed by both prokaryotes and eukaryotes, and the activity is controlled by external dissolved inorganic P concentrations and intracellular P demand (Hoppe, 2003). Natural P concentrations in the Northern parts of the Baltic are low due to relatively little anthropogenic activity and removal of inorganic P through flocculation and sedimentation (Andersson et al., 1996; Forsgren et al., 1996). The activity of the proteases and alkaline phosphatases was likely the primary drivers in generating inorganic nutrients. No difference was detected in the activities of the glucosidases or β-NAGase, which suggests that the C sources being hydrolyzed were similar in the treatments and that the added organic C did not stimulate the activities of these enzyme groups. Similarly, no difference was detected in lipase activity. This interpretation disregards the effect of time, and the variability in activities did indicate distinct enzyme responses. Extracellular enzymes vary in how their synthesis is controlled, as evident from the protease and alkaline phosphatase activities measured here, and to which environmental cues they respond (Arnosti, 2011). Moreover, they have a variable distribution and phylogenetic placement in natural bacterial communities (Elifantz et al., 2008; Zimmerman et al., 2013). It may therefore be hard to discern clear-cut responses in extracellular enzyme activities to environmental perturbations when working with natural bacterial communities, like in the present experiment.

The bacterial community composition changed in response to treatment, causing the North and South communities to separate from the two controls, which were indistinguishable. There was also a large effect of incubation on the composition patterns, evident as a relatively uniform temporal trend in all four treatments, a common observation in incubation experiments (Piquet et al., 2010). The active communities had a similar response pattern, suggesting that the DOM additions exerted selective pressure on the present (DNA) as well as the active (RNA) bacterial communities in the DOM treatments. The divergence between the North and South communities was likely driven by the different DOM stoichiometry and concentration. Changes in community composition may influence bacterial C:N:P stoichiometry due to differences in growth and cellular content, and likewise the input stoichiometry of resources influence community composition (Makino et al., 2003). Furthermore, community composition and succession may be linked to functionality (Kirchman et al., 2004; Teira et al., 2008). However, we did not observe any significant differences between North and South in the protease activities or bacterial production despite large differences in community composition. Hence, with respect to these functionalities the communities of the two treatments were functionally redundant.

To examine the communities and identify putative bacterial populations responding to DOM load, individual populations (i.e., OTUs), which contributed significantly to the differences in community composition between the treatments, were identified for the start and end of the experiment. In the day 0 communities, five Bacteroidetes populations and one Alphaproteobacteria population (*Rhodobacter*) were identified, and all except OTU_761 had the highest relative abundance in North or South. Bacteroidetes are common and widespread in marine and coastal bacterioplankton communities (DeLong et al., 1993; Cottrell and Kirchman, 2000; Kirchman, 2002). They are often linked to the degradation of high molecular weight organic matter (Covert and Moran, 2001; Gómez-Pereira et al., 2012) and enriched in carbohydrate-active hydrolases and other functions compliant with a lifestyle of utilizing high molecular weight DOM (Davey et al., 2001; Cottrell et al., 2005; Teeling et al., 2012). Most of the Bacteroidetes populations were assigned to Flavobacteriaceae, a widespread group commonly found in soil, fresh and marine waters, and many isolates are known enzyme producers (Bernardet and Nakagawa, 2006). Another Bacteroidetes was a *Fluviicola* population which dominated in the control treatments. Known *Fluviicola* strains are from freshwater environments and they utilize carbohydrates for growth (Muramatsu et al., 2012). Alphaproteobacteria also contributed to the difference between communities with a *Rhodobacter* population. These are also common members of coastal bacterioplankton and often observed as particle-colonizers (González and Moran, 1997; Crump et al., 1999). In the active communities two populations contributed to the differences between treatments. One of these populations also appeared in the total community (OTU_19) while the other population was related to *Delftia* (Betaproteobacteria). Both populations were highest in the South community. Previous studies have linked members of Betaproteobacteria, including a relative to Comamonadaceae which *Delftia* is grouped in, to the utilization of riverine DOM (Kisand and Wikner, 2003). Consequently, the DOM had dramatically altered the community structure, just 3 days after the initial DOM additions.

At the end of the experiment community compositions had changed, and the populations contributing to the differences between treatments also differed. Two populations from Gemmatimonadetes were present in the DOM treatments, with the highest abundances in North. A single population in the active communities contributed significantly to the treatment differences at the end, the Bacteroidetes population OTU_761 (*Fluviicola*). This population was the only one occurring both at the start and end, where it had increased its relative abundance in North and decreased in the two controls, suggesting it responded to the DOM. Both Gemmatimonadetes and Planctomycetes inhabit aquatic and terrestrial ecosystems (Ward et al., 2006). Gemmatimonadetes are one of the major phyla in soil communities (DeBruyn et al., 2011), and the populations occurring at the end of the experiment may well originate from the added DOM as the soil extracts were not sterile. However, estuarine communities constantly receive an inflow of terrestrial and freshwater bacteria from surrounding sources, and the brackish conditions and long water retention times (>5 years) in the Northern Baltic Sea has previously been hypothesized to promote relatively stable bacterioplankton communities of uniquely adapted bacteria (Riemann et al., 2008). Furthermore, all the populations responding to the treatments during the experiment, represent phylogenetic groups which have previously been linked to the degradation of riverine DOM (Kisand et al., 2002; Kisand and Wikner, 2003), and which are commonly identified as responsive in experiments amended with DOM (Pinhassi et al., 2004; Teeling et al., 2012; Lindh et al., 2015). Moreover, the dominance of Bacteroidetes in the GLM analysis suggests that this phylum is particularly responsive to elevated levels of DOM. Moreover, the different response patterns in OTUs contributing to the day 0 and day 28 community differences, suggest that bacterial populations may respond to the particular DOM composition, and that the continued treatment differences over time, were maintained by underlying shifts within each community.

## Conclusion

The results presented here indicate that increased DOM inflow will have a major effect on the recipient ecosystem. The DOM caused shifts in bacterioplankton community composition and stimulated protease activity and bacterial DOM consumption. Despite that the different types of DOM selected for distinct communities, only some functions responded, and these did not differ between the DOM treatments. The bacterioplankton utilization of DOM corresponded to approximately 25–85% of the supplied DOM depending on type (DON, DOP, or DOC), and the microbial activity caused a release of inorganic nutrients. This conceivably stimulated a succession in phytoplankton community composition as well as increasing biomass towards the end of the experiment. The increased phytoplankton biomass indicates that access to mineralized inorganic nutrients partially outweighs the detrimental effect of extensive light attenuation associated with the high DOM levels. Our experimental data suggest that a large fraction of future elevated DOM inflow to the Baltic Sea will be mineralized by bacterioplankton with consequences for nutrient biogeochemistry and primary production in coastal regions.

## Author Contributions

ST, OR, AA, and LR conceived the study. ST, OR, and AA carried out the experimental work. ST, NJ, HS, CS, JD, and LR performed the analyses and interpretations. ST and LR wrote the paper including the comments and revisions from all co-authors. All authors have read and approved the submitted version.

## Conflict of Interest Statement

The authors declare that the research was conducted in the absence of any commercial or financial relationships that could be construed as a potential conflict of interest.
